# Measuring Bird Morphology From Field Photographs

**DOI:** 10.1002/ece3.74422

**Published:** 2026-10-05

**Authors:** Facundo X. Palacio, Evaristo Elso, Diego Montalti

**Affiliations:** ^1^ Sección Ornitología, División Zoología Vertebrados, Facultad de Ciencias Naturales y Museo Universidad Nacional de La Plata La Plata Buenos Aires Argentina; ^2^ Consejo Nacional de Investigaciones Científicas y Técnicas La Plata Buenos Aires Argentina

**Keywords:** digital photography, morphometric measurements, photogrammetry, pinhole camera model

## Abstract

Morphometric measurements are among the most important phenotypic traits in birds. However, traditional linear measurements can be invasive, are often limited to species that are easier to capture, may be constrained in challenging environments, and typically allow for lower sampling efficiency per unit time. We applied a digital photography approach based on the “pinhole camera model” using an experimental setup with four bird species. We assessed the performance of this approach by varying elevation angles, distances to the subject, and orientations relative to the focal plane to estimate body, tarsus, wing, and bill length. We then extended this approach to 37 bird species photographed in the field and compared our estimates with measurements derived from a global database and museum specimens. We evaluated the performance of single‐species trait estimation and the ability to infer community‐level metrics that incorporate trait diversity. Under optimal viewing conditions (0° relative to the focal plane), body and wing length showed the smallest biases in the experimental setting, ranging from approximately 1%–6% and 1%–9%, respectively. Tarsus and bill length exhibited larger biases, ranging from about 5%–12% and 2%–15%, respectively. We found strong correlations between traits derived from photographic measurements and those obtained from both the global database and our own museum specimens (*ρ* = 0.82–0.95). We found no differences in functional trait diversity metrics between photograph‐based estimates and those derived from the global database, but detected significant differences in assemblage‐level metrics based on museum measurements. Our approach enables robust estimation of linear measurements from a single image, using field‐acquired data, under specific conditions. We provide practical recommendations for applying the pinhole camera model to wild birds. It is straightforward to implement and generalizable across species, thereby enhancing its applicability and expanding opportunities to use camera images in novel ways in bird ecology and behavior.

## Graphical abstract

We evaluated a digital photography approach based on the pinhole camera model to estimate bird morphometric traits from single images, testing its accuracy under controlled conditions and across 37 species photographed in the field. Photographic measurements showed strong agreement with museum and database‐derived traits (ρ = 0.82–0.95), with wing and bill length yielding the most accurate estimates. Despite some trait‐specific biases, the method reliably captured species and community‐level functional variation, providing a simple, non‐invasive, and broadly applicable tool for estimating avian morphology from field photographs.

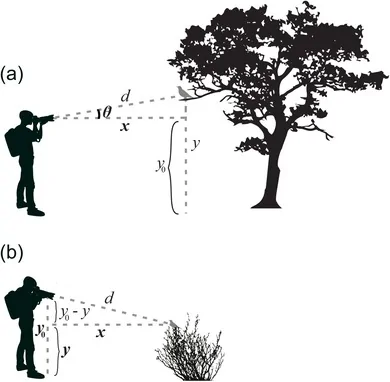

## 1 Introduction

Morphometric measurements represent one of the most important phenotypic traits measured in birds, as these underpin a wide array of ecological and evolutionary phenomena (James 1982; Pigot et al. 2020). Linear morphological traits, such as body, tarsus and bill length, have been traditionally measured from either captured individuals or museum specimens (Rising and Somers 1989; Montalti et al. 2012; Fuchs et al. 2017; Piro et al. 2018). Given their importance in bird biology, several handbooks have been dedicated to standardized measurement protocols (e.g., Baldwin et al. 1931; Dzubin and Cooch 1992; Ralph 1993; Winker 1998; Flores 1999; Burtt 2002), alongside extensive online databases that compile morphometric measures (e.g., Ricklefs 2017; Burgio et al. 2019; Rodrigues et al. 2019; Tobias et al. 2022).

Beyond these widely used classical measurements, alternative approaches to estimate morphological measurements in wildlife have been developed. For instance, camera trapping has been applied to estimate animal size directly from images in medium and large terrestrial mammals (Berger 2012; Willisch et al. 2013; Tarugara et al. 2019; Cui et al. 2020). More recently, Leorna et al. (2022) used a conceptual model in photogrammetry known as the “pinhole camera model” to estimate the size of captive Reindeer ( *Rangifer tarandus* ) using pixel measurements taken directly from an image. These authors found that the total mean relative error of estimated animal sizes in the field was around −9%, yielding accurate and precise estimates over a range of animal sizes and distances from the camera (Leorna et al. 2022). This model is appealing due to its simplicity, as it is based on proportional relationships between size and distance. Nevertheless, this approach has been tested in large terrestrial mammals only, which are expected to produce less relative bias than small species, such as bats, birds, or reptiles.

Despite these developments, photography has been insufficiently tested as a tool to replace or complement traditional manual morphometric measurements in birds. For instance, Mahendiran et al. (2018) estimated bill, head, tarsus, and total body length using digital photograph's metadata (field of view, focal length, object‐distance) under experimental (cardboard fish models and bird museum specimens) and field conditions (free‐ranging chickens). The relationships between estimated and true measurements did not differ from the 1:1 regression line, indicating accurate estimates of this approach (Mahendiran et al. 2018). However, these authors only tested the method in a single, large‐bodied species and within a limited range of distances (48 cm to 2.72 m) from the ground. In another study, Williams et al. (2020) compared tarsus and bill length, width, and depth using digital photographs and those obtained using traditional manual calipers on captured individuals. Photographs slightly overestimated measurements in all cases, indicating that both measurements may not be directly comparable (Williams et al. 2020). More recently, Tansell et al. (2026) measured kiwi bills with high accuracy (< 1.5%) under ideal conditions (daylight and stationary taxidermied specimens) using a home‐modified stereo camera. However, measurements obtained from live individuals in field conditions at night showed substantial biases (> 20%; Tansell et al. 2026). Although these studies have provided valuable insights into the magnitude of error associated with estimating morphometric measurements from bird photographs, the broader potential of photographs of wild birds as a source of morphological data remains largely underexplored.

Here, our aim is challenging—to estimate bird measurements from photographs taken in the field. The high mobility and wariness of human presence makes obtaining sharp, well‐composed images difficult. This is particularly true for passerines, who frequently exhibit rapid flight or occupy inaccessible habitats, such as dense vegetation or high canopy levels, further complicating detection and image capture. In addition, their cryptic plumage enhances camouflage, while variable lighting conditions need frequent exposure adjustments (Read 2019). However, if photographic measurements prove to be comparable to manual methods, they could offer significant advantages: (1) a non‐invasive approach that eliminates the need for capturing individuals, (2) an increased sampling area per unit of time, (3) the ability to revise data easily, (4) the ability to obtain measurements from taxa that are more difficult to capture or detect, such as raptors and parrots, (5) improved feasibility in challenging environments, including densely populated cities, and (6) the capacity to account for intraspecific variation in methods of community ecology that require sampling traits of the same individuals observed within assemblages. In this study, we tested whether the “pinhole camera model” can produce reliable estimates of morphometric measurements under a set of experimental and field conditions. To this end, we used museum‐mounted bird specimens to test the effects of different distances and angles on morphometric measurements, and field measurements to recover trait‐based community metrics.

## 2 Materials and Methods

### 2.1 Experimental Setting

The “pinhole camera model” describes the relationship between a three‐dimensional scene and its two‐dimensional projection on the camera sensor. It relies on simple trigonometry, where light from a point in the physical scene passes through the camera's pinhole or aperture and is mapped to a corresponding point on the sensor (Kannala et al. 2008; see Figure 1 in Leorna et al. 2022). The relationship between a physical object and its projection on an image is summarized by the proportional relationship

$$\frac{S_{o}}{d}=\frac{S_{i}}{f}$$

where *S*_*o*_ is the size of the object (physical dimension), *S*_*i*_ is the size of the object on the image, *d* is the distance to the object, and *f* is the focal length. To test the “pinhole camera model” under controlled situations, an experimental design was set using museum‐mounted specimens to simulate birds in lifelike positions. Four common bird species in the Neotropics spanning a range of body sizes (~25–300 g) were selected: House Sparrow *Passer domesticus* (Passeridae), Rufous‐bellied Thrush *Turdus rufiventris* (Turdidae), Campo Flicker *Colaptes campestris* (Picidae), and Chimango Caracara *Milvago chimango* (Falconidae) (Museo de La Plata, Argentina). The specimens were photographed using a camera (Canon T2i) with a 12 MP sensor and a Canon 70–300 mm F/4–5.6 lens from various heights *y*, horizontal distances *x* and angles relative to the focal plane *α* (Figure 1a). The camera was mounted on a tripod at a fixed height of 1.50 m. Eleven height *y* levels were considered, corresponding to the ground plus eleven steps of a foldable aluminum articulated ladder 3.50 m in length. The ladder was positioned against a straight tree trunk with a 10° inclination at its base. The exact height of each level was calculated by summing the step height and the base height of the mounted specimen. At each step, photographs were taken from four different angles relative to the focal plane *α* (0°, 20°, 40°, and 60°; Figure 1b). We limited the angle to a maximum of 60°, as size estimates become highly unreliable beyond this threshold (Leorna et al. 2022). In our dataset, images with angles exceeding 60° obscured the bill almost entirely (Figure 1b). Each specimen was positioned over the image of a protractor to precisely define the different angles. Specimens were rotated around an axis approximately located at the center of the body, with the longitudinal axis of the specimen initially aligned parallel to the focal plane (0°). Subsequent orientations were obtained by rotating the specimen away from the focal plane by 20°, 40°, and 60°, while maintaining the same camera position. Therefore, the angle represents the rotation of the specimen relative to the focal plane, rather than the exact orientation angle of each individual morphological trait (see Discussion). Photographs were captured from three fixed horizontal distances *x*: 3.0, 5.0, and 7.0 m. These distances were selected to match common distances in bird observations (see Results). Horizontal distances were adjusted by shifting a measuring tape perpendicular to the vertical plane of the ladder as height increased. The design was replicated for each of the four species, covering all combinations of heights, angles, horizontal distances, and species, resulting in a total of 580 photographs (576 combinations plus 4 photographs including a metric scale reference). The reference photographs were measured using ImageJ 1.4 g (Schneider et al. 2012), and the resulting measurements were used as the reference values for each morphological trait against which photogrammetric estimates were compared. All the images were stored in RAW format. With this information, we estimated the distance to the bird *d* as *d* = (*x* ^2^ + (*y*—1.50)^2^)^1/2^ and the elevation angle of the bird relative to the ground *θ* as *θ* = arctan(*y*/*x*) (if *y* ≤ 1.50 m) or *θ* = arctan ((*y*—1.50)/*x*) (if *y* > 1.50 m). This angle ranged between 0.08° and 34.22° as a result of fixing the distance to the bird and the height. We estimated object distance directly in the field rather than relying on EXIF metadata, as this information was not provided by our camera model, it is not consistently recorded across all cameras and lenses, and when available, it may be unreliable (Mahendiran et al. 2018).

**FIGURE 1:**
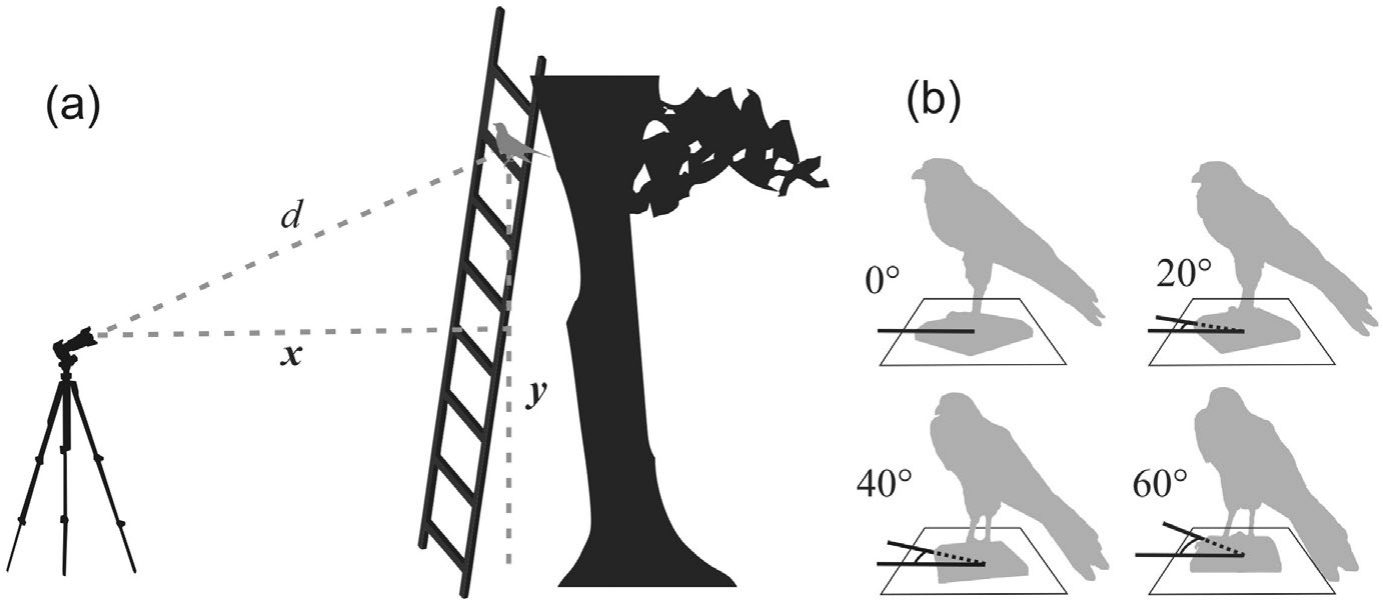
Experimental setup for photographing mounted specimens. (a) Camera positioning relative to the specimens. (b) The four specimen orientations with respect to the focal plane (0°, 20°, 40°, and 60°). *d* = distance between the camera and the bird; *x* = horizontal distance; *y* = height.

### 2.2 Field Tests

To test the performance of the “pinhole camera model” under realistic field conditions, we recorded images of wild birds in 10 field surveys between 18 July and 19 November, 2024 (Austral winter and spring) in forests from NE Buenos Aires province, Argentina. Transects were conducted within the first 3 h after sunrise in two habitat types: a native forest and one invaded by Glossy Private ( *Ligustrum lucidum* ) in “El Destino” private reserve (35° 09′ S, 57° 02′ W), located within the Biosphere Reserve “Parque Costero del Sur”. The native forest consists of xeromorphic forests, locally known as “talares,” which develop on calcareous soil deposits along the shore of the Río de La Plata (Goya et al. 1992). These forests are embedded within a grassland matrix, contributing to approximately 10% of the total forest cover. The dominant native tree species are Spiny Hackberry ( *Celtis tala* ), “Coronillo” (*Scutia buxifolia*), “Molle” (*Schinus longifolia*) and “Sombra de Toro” (*Jodina rhombifolia*) (Goya et al. 1992). The invaded area consists of a nearly monospecific forest dominated by *Ligustrum lucidum* , reaching up to 20 m in height. However, some sections also host other exotic tree species, including *Eucalyptus* sp., *Fraxinus* sp., *Cupressus*, *Platanus* sp., *Populus* sp., and *Quercus* sp. (Franco et al. 2018).

In the “talares”, we conducted an intensive search on forest patches during three continuous hours, walking slowly and taking photographs of individual birds in a 20 m‐wide transect (Palacio et al. 2019). Given the high density of trees and saplings in the invaded forest, we adopted a similar approach in this habitat type but using trails to avoid bird disturbance. For each photographed individual, we recorded the horizontal distance *x* to the bird and the elevation angle *θ* using a measuring tape and a clinometer, respectively (Figure 2a). For practical reasons, if the bird was located at a lower height than that of the camera *y*_0_, its height *y* was measured directly with the tape instead of the angle (Figure 2b). Then, the distance to the bird *d* was estimated by simple trigonometry as *d* = *x*/*cosθ* (if *y* > *y*_0_) or *d* = (*x* ^2^ + (*y*_0_ *—y*)^2^)^1/2^ (if *y ≤ y*_0_) (Figure 2). Birds photographed in the field were not captured for direct morphometric measurements, as obtaining standardized photographs and subsequently capturing the same free‐ranging individuals would be highly unfeasible, particularly for passerines. Instead, measurements obtained from field photographs were evaluated against independent reference datasets, including species‐level values from a global database and measurements that we collected from museum specimens representing the same species (see below).

**FIGURE 2:**
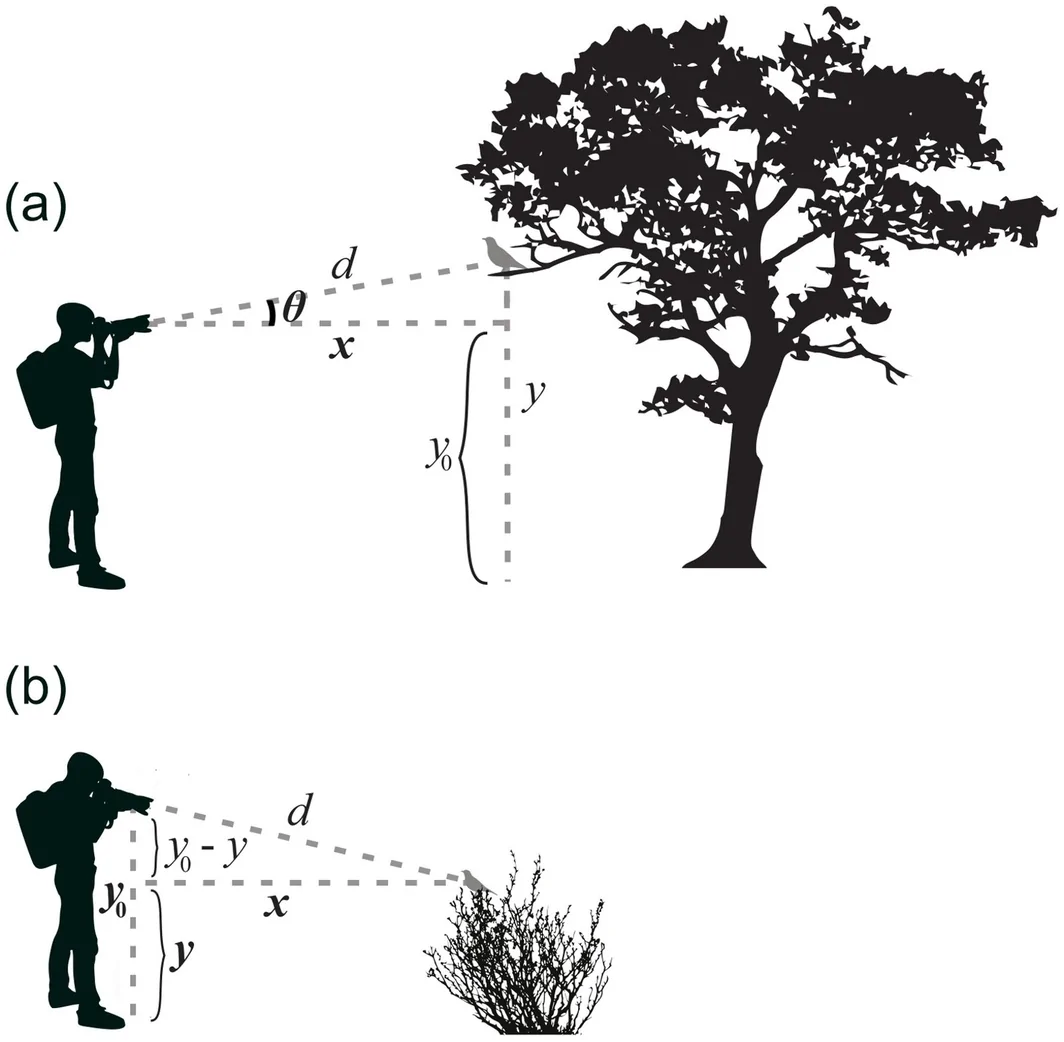
Data obtained from bird photographs in the field. When the bird was positioned higher than the camera, both the horizontal distance to the bird and the angle relative to the ground were recorded (a). When the bird was positioned lower than the camera, its height was measured directly (b). *d* = distance between the camera and the bird; *x* = horizontal distance; *θ* = angle relative to the ground; *y* = bird height; *y*_0_ = camera height.

### 2.3 Morphometric Measurements

For each photograph, we measured four (whenever possible in the case of wild birds) linear morphological traits widely used in bird ecology and evolution (e.g., Pigot et al. 2020; Tobias et al. 2022): bill length, wing length, tarsus length and body length. The first three traits were measured following Baldwin et al. (1931) and the recommendations given by Winker (1998), whereas body length was estimated by following the silhouette of the culmen‐to‐tail length (Leorna et al. 2022; Figure S1). The latter measurement differs from the typical total length used in museum and live specimens, which are taken from the tip of the bill to the tip of the tail while the bird is thoroughly relaxed, lying flat on its back, with the head bent dorsally (Baldwin et al. 1931). Therefore, this should be considered an approximation of body length. All photographs of a given species were measured by the same observer to minimize potential biases from inter‐observer variability. We then estimated each physical dimension as (Leorna et al. 2022)

$$S_{o}=\frac{S_{i}d}{f}$$

where *S*_*o*_ is the size of the bird, *S*_*i*_ is the size of the bird on image (in mm), *d* is the distance to the bird (in mm), and *f* is the focal length (in mm). The focal length of each picture was retrieved from the EXIF metadata (Mahendiran et al. 2018). In contrast to Leorna et al. (2022) who converted cm to pixels as the unit of measurement, we worked directly with mm as it is a simpler and more straightforward approach. Specifically, *S*_*i*_ is the projected length of the structure on the camera sensor (mm) estimated as:

$$S_{i}=l_{\mathrm{px}}\frac{H_{\text{sensor}}}{H_{\text{image}}},$$

where *l*_*px*_ is the measured length of the structure in the digital image (pixels), *H*_*sensor*_ is the physical height of the camera sensor (14.9 mm in our camera model), and *H*_*image*_ is the height of the digital image (3465 pixels in our camera model).

Because morphometric measurements were obtained from two‐dimensional images, it was not possible to ensure that all structures were perfectly aligned with the image plane, which can introduce systematic underestimation due to specimen tilt in three‐dimensional space. To estimate body orientation, the body axis was defined by manually selecting two anatomical landmarks (*locator* function in base R): the bill tip and the base of the tail. The inclination angle *α* of this axis relative to the focal plane was calculated as follows:

$$\alpha =\arctan\left(\frac{∆y}{∆x}\right)$$

where Δ𝑥 and Δ*y* are the horizontal and vertical differences between landmarks. Under a simple projection model, the observed length corresponds to the projection of the true length onto the image plane. Thus, corrected morphometric values were obtained as *S*_*o*_/cos (*α*). This trigonometric correction assumes tilt occurs primarily along the body axis and reduces bias associated with out‐of‐plane orientation. In several cases, we could take up to three photographs of the same individual (*n* = 12 individuals). For these cases, we averaged their trait values. To assess the repeatability of these photographs of the same individuals, we computed the intraclass correlation coefficient (ICC) by fitting linear mixed‐effects models for each trait using individual identity as a random effect. Repeatability was high for body length (ICC = 0.94), wing length (ICC = 0.81), and bill length (ICC = 0.80), whereas tarsus length showed comparatively lower repeatability (ICC = 0.73), consistent with the greater sensitivity of this trait to changes in viewing angle observed in the experimental analyses (see Discussion). All photographs were measured using the software ImageJ 1.4 g (Schneider et al. 2012), using the segmented line tool for body length and the straight‐line tool for the rest of measurements in pixels (Leorna et al. 2022).

### 2.4 Data Analysis

Our analysis of the experiment was based on how four main factors influenced size measurements: the bird‐observer distance *d*, the angle of the bird relative to the focal plane *α*, the elevation angle between the observer and the bird relative to the ground *θ* and the size of the species. For each species, we inspected which traits and angles produced values farther away from the “true value” (i.e., accuracy), and which produced more variability in size estimates (i.e., precision = standard deviation of the absolute percentage bias across measurements). We then fitted a set of multiple linear regression models for each species (*n* = 144 photographs per species) between the absolute relative bias of each measurement (100 × |observed value – “true” value|/“true” value; response), the bird‐observer distance, the angle of the bird relative to the focal plane (continuous), and the elevation angle (predictors). We used the absolute bias instead of the raw bias, as we were interested in how bias changed with predictors, regardless of its direction. Because residual diagnostics revealed heteroscedasticity, we fitted generalized least squares (GLS) models incorporating a power variance function of the angle relative to the focal plane to account for non‐constant error variance. Potential collinearity among predictors was assessed using variance inflation factors. These ranged from 1.00 to 1.18 across all models, indicating negligible collinearity among predictors.

To assess the validity of the method in the field, we compared photograph estimates from morphological data retrieved from AVONET (Tobias et al. 2022). It should be noted that these measurements do not represent the “true” size of birds, but an approximation. Because AVONET includes data from individuals across their entire distribution, potentially encompassing high intraspecific variation (Palacio et al. 2025), we repeated the analysis using only individuals from the same geographic area. To this end, we measured body, bill, tarsus and wing length on 2–5 specimens (adults, males and females) from 37 species housed at the Ornithology collection of Museo de La Plata (La Plata, Argentina) with a digital caliper (nearest 0.01 mm). Therefore, the only difference in traits between both datasets is that body length data are not available in AVONET. To assess whether the observed regression line differed significantly from the expected 1:1 relationship (intercept = 0, slope = 1), we used a Wald test with the *linearHypothesis* function from the car package in R. In addition to testing the method with individual measurements, we evaluated its applicability for inferring community assembly patterns. Specifically, we computed Spearman's correlations between species averages obtained from field estimates and those derived from AVONET and our own measurements. A strong positive correlation, despite potential under‐ or overestimation by the method, would suggest that the relative ranking of species is preserved, with little impact on functional diversity metrics (provided intraspecific trait variation is sufficiently large). For each trait, we calculated the mean absolute percentage error (MAPE) between photographic and reference species means. For illustrative purposes, we combined all individuals from every species into a single bird assemblage and compared functional diversity metrics with those derived from simulated assemblages generated using AVONET and our measurements. We randomly sampled individuals with replacement, matching the number of individuals per species observed in the assemblage. We repeated this process to create a distribution of 500 bootstrap assemblages. For each assemblage, we built a functional trait space by performing a PCA on the trait matrix and computed functional richness and divergence with the R package *funspace* (Carmona et al. 2024). Functional richness represents the amount of functional space occupied by the assemblage, whereas functional divergence is the average distance of the data points from the centroid of the trait space (Palacio 2023). The two metrics are scale‐invariant as they measure size and relative position, respectively, meaning they remain unaffected by proportional shifts in trait values. The resulting 500 values per metric were compared with the observed functional diversity metrics to compute *p*‐values.

## 3 Results

We observed substantial variation in both accuracy and precision across species and traits in the mounted specimens (Figure 3, Table S1). Under optimal viewing conditions (0° relative to the focal plane), body and wing length exhibited the lowest biases (1%–6% and 1%–9%, respectively), whereas tarsus and bill length showed comparatively larger biases (5%–12% and 2%–15%, respectively). The magnitude of bias varied among species, and no consistent pattern emerged across body sizes. For all four species, the smallest biases generally occurred at viewing angles between 0° and 20°, whereas larger angles produced increasingly biased estimates. Detailed bias and precision estimates for each species, trait, and angle are provided in Table S1.

**FIGURE 3:**
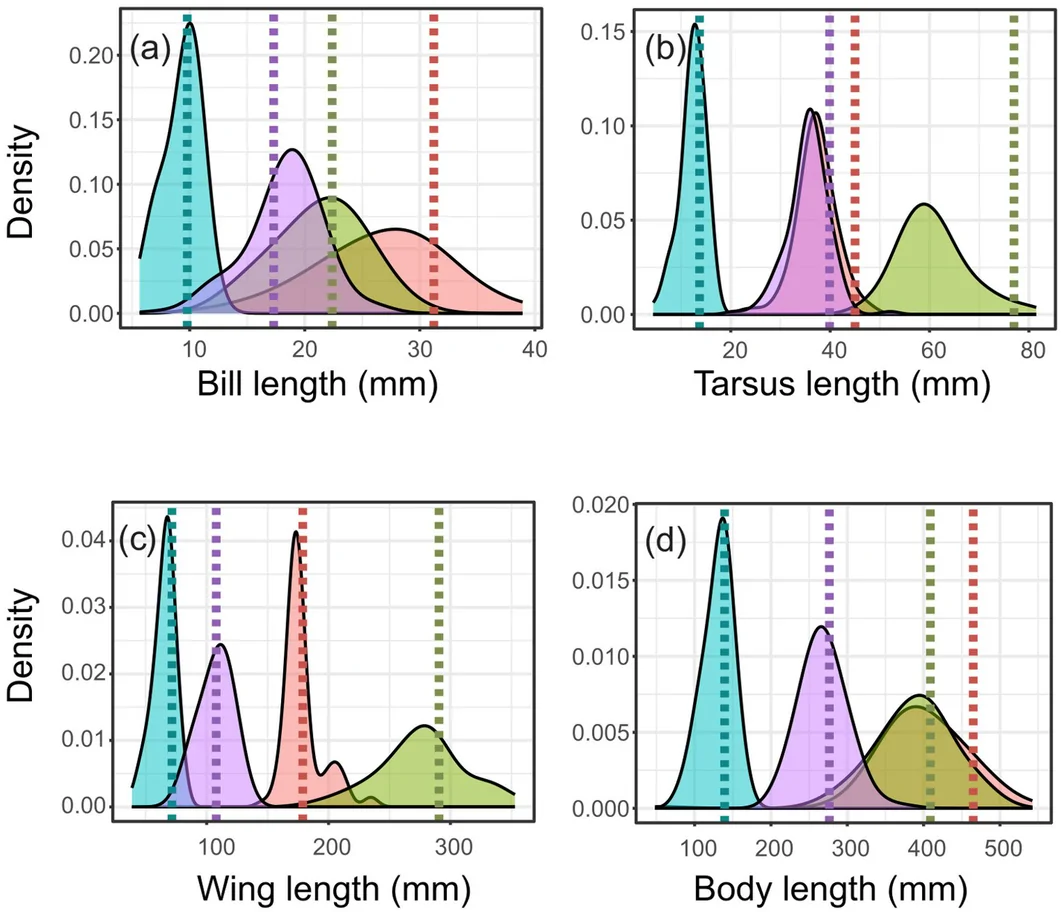
Bias in bird trait estimates obtained from mounted specimens. Curves show kernel density distributions based on photographic measurements (a: Bill length, b: Tarsus length, c: Wing length, d: Body length), while dashed lines indicate the “true” values derived from photographs including a metric scale reference. Light blue: *Passer domesticus* , violet: *Turdus rufiventris* , pink: *Colaptes campestris* , green: *Milvago chimango* .

Overall variation explained by the three predictors was very high (pseudo‐*R* ^2^ = 0.974–0.995), suggesting that these factors accounted for most of the variability in morphometric estimates. Across species and traits, the angle relative to the focal plane was the strongest predictor of measurement error, with larger angles generally producing greater bias (Table 1). Elevation angle also increased bias for several traits, although its effects were less consistent than those of the viewing angle. In contrast, distance to the camera had comparatively weak and variable effects, with significant relationships detected only for a subset of species–trait combinations (Table 1). Estimated coefficients ranged from −2.87 to 2.91, corresponding to an approximate change in relative bias of 3% per meter. Application of the cosine correction altered the magnitude and direction of measurement bias across species, traits, and angles, with the overall effect varying among morphological measurements (Table S2). Because multiple hypothesis tests were performed across species, traits, and predictors, individual *p*‐values close to the conventional significance threshold should be interpreted with caution. Our conclusions are based on the overall consistency of the observed patterns rather than on isolated borderline significant results.

**TABLE 1:** Results of different factors (distance to the bird, angle relative to the focal plane, and elevation angle) on relative bias of bird measurements (tarsus length, body length, bill length and wing length) across mounted specimens of four bird species.

Species	Trait	Parameter	Estimate	Standard error	*t*	*p*
*Passer domesticus*	Tarsus length	Intercept	3.576	4.031	0.887	0.377
		Distance to the bird	0.625	0.605	1.033	0.303
		**Angle (focal plane)**	**0.255**	**0.039**	**6.584**	**< 0.0001**
		**Angle (elevation)**	0.123	0.122	1.010	0.314
	Body length	**Intercept**	**6.575**	**2.211**	**2.974**	**0.003**
		**Distance to the bird**	**−1.107**	**0.335**	**−3.309**	**0.001**
		**Angle (focal plane)**	**0.250**	**0.024**	**10.504**	**< 0.0001**
		**Angle (elevation)**	**0.312**	**0.067**	**4.624**	**< 0.0001**
	Bill length	**Intercept**	**6.996**	**2.310**	**3.029**	**0.003**
		Distance to the bird	−0.601	0.349	−1.721	0.088
		**Angle (focal plane)**	**0.256**	**0.025**	**10.266**	**< 0.0001**
		**Angle (elevation)**	**0.157**	**0.071**	**2.213**	**0.029**
	Wing length	**Intercept**	**5.037**	**1.747**	**2.882**	**0.005**
		**Distance to the bird**	**−0.608**	**0.266**	**−2.291**	**0.023**
		**Angle (focal plane)**	**0.279**	**0.022**	**12.851**	**< 0.0001**
		**Angle (elevation)**	**0.205**	**0.054**	**3.834**	**< 0.0001**
*Turdus rufiventris*	Tarsus length	Intercept	−5.455	2.870	−1.901	0.059
		**Distance to the bird**	**2.114**	**0.420**	**5.037**	**< 0.0001**
		Angle (focal plane)	−0.004	0.028	−0.141	0.888
		**Angle (elevation)**	**0.611**	**0.087**	**7.032**	**< 0.0001**
	Body length	**Intercept**	1.934	2.361	0.819	0.414
		Distance to the bird	0.152	0.341	0.447	0.656
		**Angle (focal plane)**	**0.156**	**0.024**	**6.573**	**< 0.0001**
		Angle (elevation)	0.105	0.068	1.539	0.126
	Bill length	**Intercept**	**17.246**	**3.900**	**4.422**	**< 0.0001**
		**Distance to the bird**	**−1.428**	**0.582**	**−2.453**	**0.015**
		Angle (focal plane)	0.029	0.037	0.790	0.431
		**Angle (elevation)**	**0.413**	**0.116**	**3.556**	**0.001**
	Wing length	**Intercept**	**15.073**	**2.447**	**6.159**	**< 0.0001**
		**Distance to the bird**	**−1.503**	**0.363**	**−4.146**	**< 0.0001**
		**Angle (focal plane)**	**0.106**	**0.023**	**4.582**	**< 0.0001**
		Angle (elevation)	−0.038	0.072	−0.520	0.604
*Colaptes campestris*	Tarsus length	**Intercept**	**13.645**	**3.986**	**3.423**	**0.001**
		**Distance to the bird**	**1.353**	**0.477**	**2.839**	**0.006**
		**Angle (focal plane)**	**−0.143**	**0.052**	**−2.735**	**0.008**
		**Angle (elevation)**	**0.247**	**0.091**	**2.713**	**0.008**
	Body length	Intercept	4.457	2.594	1.718	0.088
		Distance to the bird	0.414	0.396	1.045	0.298
		**Angle (focal plane)**	**0.109**	**0.027**	**4.085**	**< 0.0001**
		**Angle (elevation)**	**0.360**	**0.076**	**4.754**	**< 0.0001**
	Bill length	**Intercept**	**16.840**	**3.429**	**4.911**	**< 0.0001**
		**Distance to the bird**	**−1.912**	**0.520**	**−3.678**	**< 0.0001**
		**Angle (focal plane)**	**0.288**	**0.033**	**8.609**	**< 0.0001**
		Angle (elevation)	−0.129	0.098	−1.308	0.193
	Wing length	**Intercept**	**21.142**	**1.789**	**11.815**	**< 0.0001**
		**Distance to the bird**	**−2.435**	**0.269**	**−9.059**	**< 0.0001**
		**Angle (focal plane)**	**−0.056**	**0.017**	**−3.276**	**0.001**
		Angle (elevation)	−0.035	0.051	−0.680	0.498
*Milvago chimango*	Tarsus length	**Intercept**	**26.010**	**3.158**	**8.237**	**< 0.0001**
		**Distance to the bird**	**−2.072**	**0.464**	**−4.466**	**< 0.0001**
		**Angle (focal plane)**	**−0.065**	**0.032**	**−2.031**	**0.044**
		**Angle (elevation)**	**−0.334**	**0.090**	**−3.732**	**< 0.0001**
	Body length	**Intercept**	**5.247**	**2.518**	**2.083**	**0.039**
		Distance to the bird	−0.738	0.386	−1.910	0.058
		**Angle (focal plane)**	**0.219**	**0.026**	**8.565**	**< 0.0001**
		**Angle (elevation)**	**0.273**	**0.075**	**3.625**	**< 0.0001**
	Bill length	**Intercept**	**10.586**	**3.598**	**2.942**	**0.004**
		**Distance to the bird**	−0.344	0.529	−0.650	0.517
		**Angle (focal plane)**	**0.232**	**0.036**	**6.376**	**< 0.0001**
		Angle (elevation)	−0.198	0.107	−1.854	0.066
	Wing length	Intercept	2.359	2.553	0.924	0.357
		Distance to the bird	−0.126	0.390	−0.322	0.748
		**Angle (focal plane)**	**0.197**	**0.025**	**7.733**	**< 0.0001**
		**Angle (elevation)**	**0.283**	**0.076**	**3.732**	**< 0.0001**

*Note:* Results of generalized least squares models are shown. Significant *p*‐values are in bold with *α* = 0.05.

In the field, we captured a total of 115 photographs belonging to 99 individuals from 37 species. Mean bird‐observer distance was 9.52 ± 6.16 m (median = 7.80 m), ranging between 1.55 ( *Phylloscartes ventralis* ) and 39.03 m ( *Leptotila verreauxi* ), whereas mean bill‐tail angle was 38.45° ± 22.5° (median = 44.18°). The estimated measurements were highly correlated with those derived from AVONET, and ranged between 0.84 (tarsus length) and 0.92 (bill length) (*n* = 37, all p's < 0.0001). Similarly, photograph measurements were highly correlated with museum measurements, ranging between 0.82 (tarsus length) and 0.95 (body length) (*n* = 37, all p's < 0.0001). However, most estimates (without the trigonometric correction) fell below the 1:1 line for all traits, preserving the relative size (Figure 4). In particular, wing length showed the largest deviations from the 1:1 line for both datasets, particularly for large (> 100 mm) wings (*F* = 45.19, gl_N_ = 36, gl_D_ = 34, *p* < 0.0001; Figure 4), whereas tarsus length showed the lowest deviations (*F* = 5.90, gl_N_ = 17, gl_D_ = 15, *p* = 0.01). At the species‐mean level (*n* = 37 species), photographic estimates showed varying levels of agreement with museum measurements and AVONET values. Mean absolute percentage errors ranged from 15.38 (body length) to 20.38% (bill length) for comparisons with museum measurements and from 17.09 (tarsus length) to 34.96% (bill length) for comparisons with AVONET (Table S3). Angle‐corrected measurements showed closer agreement with the 1:1 line on average, but greater variability around it (Figure S2).

**FIGURE 4:**
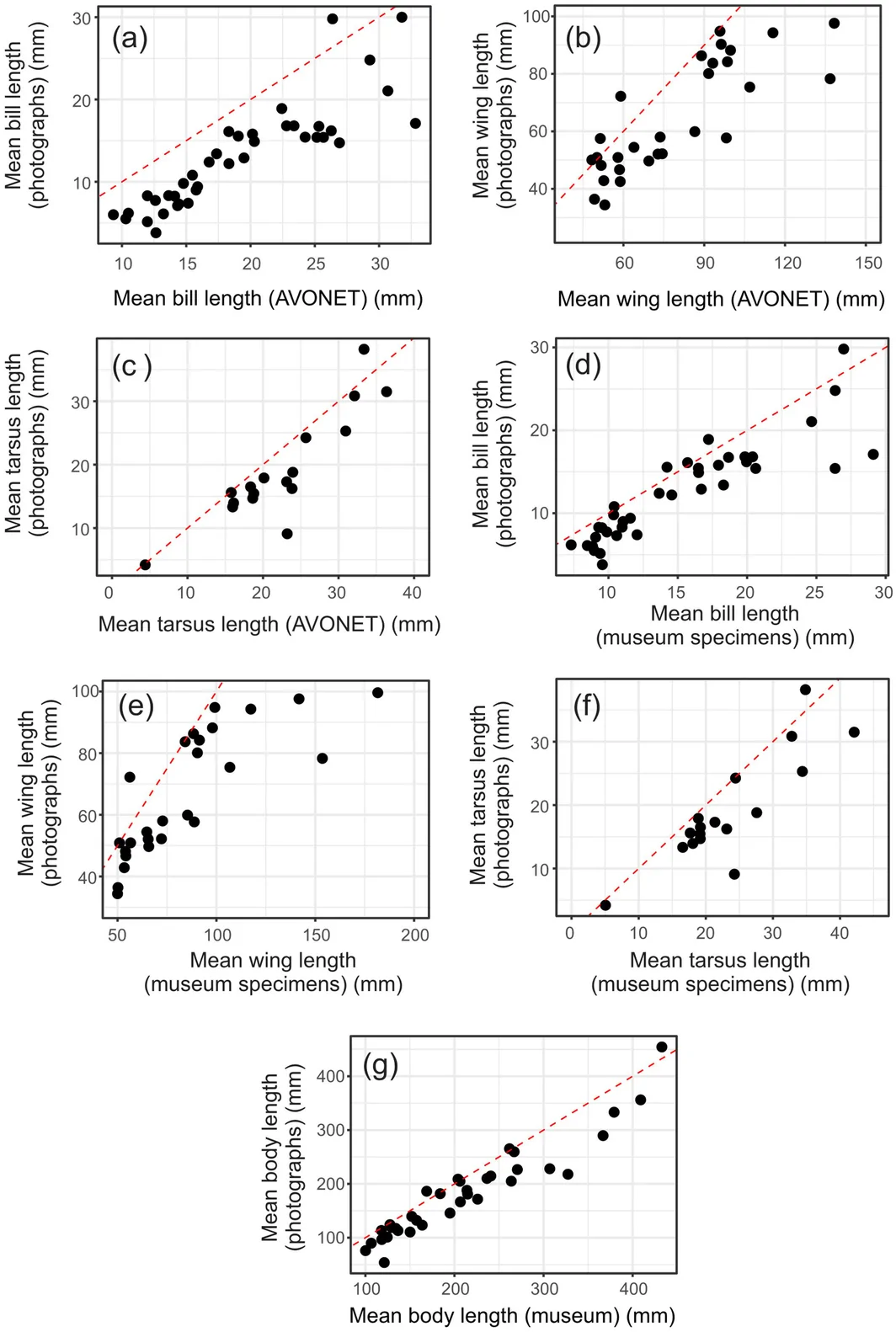
Correlations between (angle‐uncorrected) measurements obtained from photographs and those from AVONET (a–c) and museum specimens (d–g). Points represent species. Dashed lines indicate the 1:1 relationship.

When inspecting assemblage‐level patterns using metrics derived from AVONET, both observed functional richness (FRic = 32.74) and divergence (FDiv = 0.64) fell within the distribution of both metrics derived from AVONET (mean FRic = 31.69, SE = 2.13, *p* = 0.312; FDiv = 0.68, SE = 0.03, *p* = 0.688) (Figure 5a,b). By contrast, observed functional richness (FRic = 46.81) was significantly higher than assemblage‐level metrics based on museum measurements (mean FRic = 42.95, SE = 1.05, *p* < 0.001), whereas observed functional divergence (FDiv = 0.50) was significantly lower (FDiv = 0.53, SE = 0.01, *p* < 0.001), likely because both metrics derived from museum specimens showed low variance (Figure 5c,d).

**FIGURE 5:**
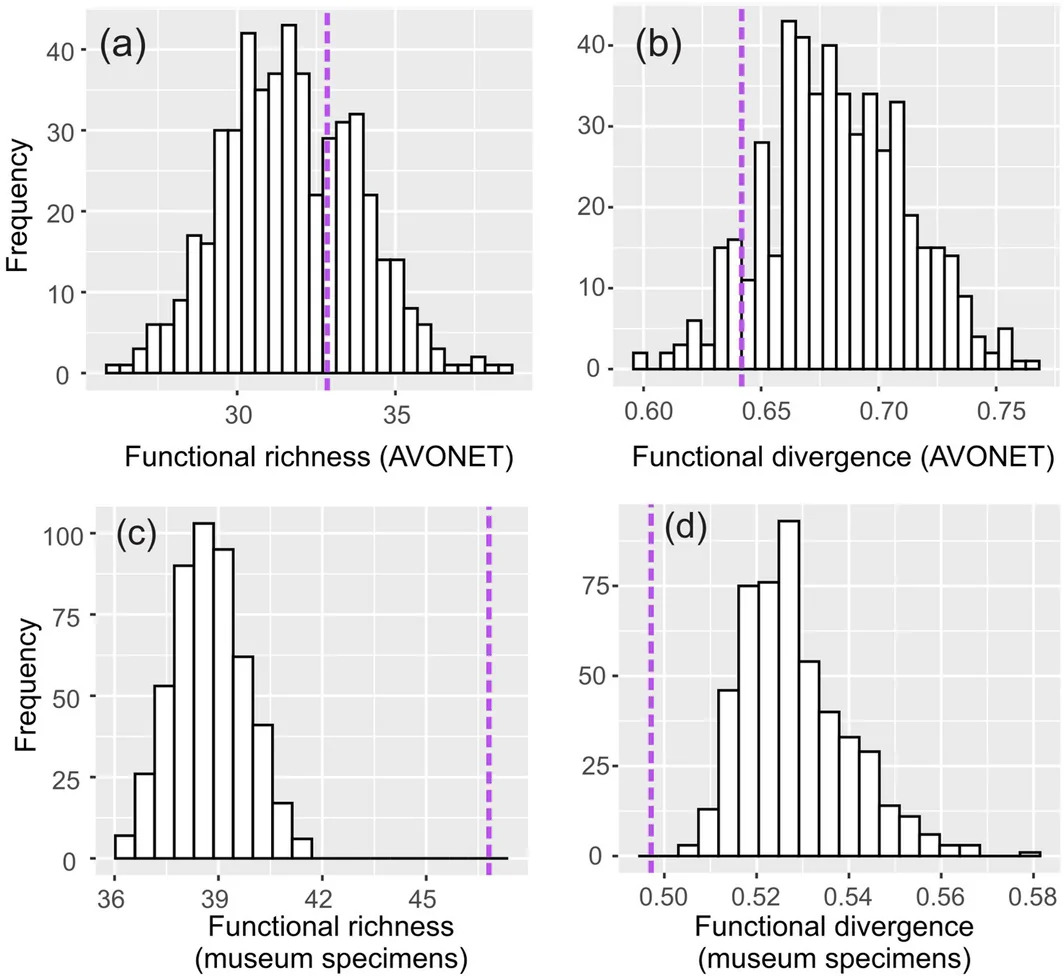
Comparison of functional diversity metrics (richness and divergence) derived from field photographs, AVONET (a–b) and museum specimens (c–d). Histograms show the distribution of 500 bootstrap simulation replicates, whereas the dashed line indicates the observed functional diversity from field photographs (see Methods).

## 4 Discussion

Linear morphological measurements in birds have traditionally been obtained through the capture of living individuals or from museum specimens. However, estimating morphometric traits from photographs offers a non‐invasive alternative with the potential to expand research in novel directions. Among its main advantages are the ability to obtain measurements from taxa that are difficult to capture or closely approach, such as raptors and parrots, as well as its applicability in challenging environments, including densely populated urban areas where traditional sampling is often limited or risky. Here, we used an experimental setup in which we manipulated viewing angle relative to the focal plane, elevation, and distance to the observer. We applied this design to four bird species differing in body size and quantified four morphological traits (body, tarsus, wing, and bill length). We then evaluated how measurements derived from photographs using the pinhole camera model (Leorna et al. 2022) affect relative bias and identified the factors that contribute most strongly to this bias. Our results show that wing and bill length can be reliably estimated from bird photographs, with relative underestimations of approximately 1%–10% regardless of species size. In contrast, body and tarsus length exhibited larger biases (ca. 3%–17%). This pattern is expected as the wing and bill are typically fully exposed when birds are perched, facilitating more accurate measurements. By contrast, tarsus length is more difficult to quantify because the joint with the tibia is often covered by feathers, requiring the observer to approximate its position. Estimating body length is also more challenging, as it must be measured from a natural, often curved posture in photographs, forcing the observer to follow the bird's irregular outline. Accordingly, we recommend prioritizing bill or wing length as more reliable traits for photographic measurement in this context.

The factor that most contributed to relative bias in morphological measurements was the angle of the bird relative to the focal plane, and to a lesser extent, the elevation angle. In contrast, the distance to the bird had almost a negligible effect on bird measurements. In a three‐dimensional context, body orientation can be decomposed into yaw, pitch, and roll, corresponding to rotations around the vertical, lateral, and longitudinal axes, respectively. However, accurately estimating these components from a single two‐dimensional image is inherently difficult because perspective projection collapses depth information. As a result, different combinations of yaw and pitch can produce indistinguishable projections in the image plane, leading to ambiguity in the true spatial orientation of the subject. While roll (rotation within the image plane) can be directly quantified from visible landmarks, yaw and pitch involve out‐of‐plane rotations that cannot be uniquely resolved without additional information (e.g., multiple views, camera calibration, or known reference geometry). This limitation constrains the ability to fully reconstruct three‐dimensional posture from standard photographic data and introduce uncertainty when interpreting morphometric measurements derived from 2D images (Hartley and Zisserman 2003). In our experimental setting, we quantified a single component of body orientation: the angle relative to the focal plane (interpreted as yaw), which is expected to be a major driver of variation in projected trait length. Because the measured structures (e.g., tarsus and bill) are approximately cylindrical or conical, rotation around their longitudinal axis (roll) is not expected to substantially affect their projected length under ideal geometric conditions. Although minor deviations from perfect symmetry or landmark definition may introduce small amounts of variability, foreshortening effects are predominantly governed by out‐of‐plane rotations (yaw and pitch), with roll contributing only second‐order effects. In contrast to cylindrical or conical structures, bird wings are better approximated as flattened, anisotropic surfaces (i.e., thin plates) with some degree of curvature. As a result, their projected dimensions are sensitive not only to out‐of‐plane rotations (yaw and pitch) but also to roll, which can substantially alter the visible extent of the wing and introduce additional variability due to partial occlusion and shape deformation. In contrast, the elevation angle have a small effect on bird measurements. Although our correction accounts for orientation‐driven foreshortening, variation in subject distance relative to the camera may introduce additional perspective effects. Because different parts of the body can lie at slightly different depths, projected lengths may deviate from the ideal cosine relationship even when yaw and pitch are minimal. However, this effect is expected to be small when object size is negligible relative to camera distance. These results highlight that measurement bias in photographic data is not uniform across traits or species, but depends on the interaction between viewing geometry and morphological characteristics. The negative relationships observed between distance to the camera and relative bias may be also explained by geometric and sampling effects inherent to photographic measurements. As the distance between the subject and the camera increases, the imaging process more closely approximates an orthographic projection, thereby reducing perspective distortions caused by depth differences within the object. In contrast, at shorter distances, even small variations in depth along the body axis can lead to greater deviations from the ideal cosine relationship, increasing measurement bias. Together, these factors suggest that increasing distance can, to some extent, reduce projection‐related bias, highlighting a trade‐off between geometric distortion and image resolution in photographic measurements. Consequently, intermediate distances likely represent an optimal compromise, minimizing projection bias while maintaining sufficient detail for precise measurements. Taking these considerations into account, we recommend that, whenever possible, morphometric measurements from photographs should be obtained when birds are approximately parallel to the focal plane (i.e., yaw within ~0°–20°), with low elevation angles (within ~0°–20°), and at relatively moderate distances from the observer (ca. 3–10 m). Although these conditions cannot always be achieved in the field, they are often attainable for certain taxa, such as shorebirds or ground‐foraging species, where individuals are more likely to be observed in suitable orientations and positions for accurate measurement. Small birds, such as passerines, are often highly mobile and typically occur under tree cover. In these cases, we recommend taking 2–3 photographs whenever possible and averaging the resulting trait estimates to improve accuracy.

The pinhole camera model opens new opportunities not only for the study of bird morphology, but also for broader questions in ecology and behavior. For instance, photographs can provide valuable information on the size of prey items consumed, such as arthropods and fruits. For example, Gaglio et al. (2017) used digital photography to investigate the diet delivered to chicks of a prey‐carrying seabird (Greater Crested Tern *Thalasseus bergii*) and compared photo‐based estimates with a traditional method based on regurgitations. Although their approach achieved high accuracy (< 1 mm) and precision (~0.5 mm), size estimates relied on using a mean bill length to infer the size of anchovies (European Anchovy). While effective in this specific case involving a single predator and prey species, this approach does not account for intraspecific variation in bill size, which may limit its broader applicability. In the context of breeding biology, the pinhole camera model can be used to quantify nest architecture from photographs. For species that build conspicuous nests but are difficult to access due to their placement (e.g., Rufous Hornero *Furnarius rufus* ; Mentesana et al. 2024), this approach would allow the characterization of key structural features such as nest diameter, height, and entrance size without the need for direct contact. Given that nest photography is generally considered non‐invasive and seems to have negligible effects on predation risk or parental feeding rates (Tan et al. 2022), this method offers a valuable tool for studying nest traits. More broadly, it opens new possibilities for linking nest architecture with environmental conditions, reproductive strategies, and fitness outcomes. In community ecology, the model enables the quantification of functional traits across broad taxonomic and geographic scales without the need for capture, as well as the capacity to account for intraspecific variation (Palacio et al. 2025). For instance, when describing functional traits such as body size, foraging stratum, and foraging behavior, linear measurements can be obtained from the same individuals while they forage, thereby capturing intraspecific variation—information that would be difficult to obtain because individuals are hard to capture while actively foraging, and capture itself would alter their behavior (Adams et al. 2011; Schlicht and Kempenaers 2015) or produce systematic sampling biases (Camacho et al. 2017). The photogrammetry model thus provides a simple and effective approach to obtain this information in the field. Indeed, we found strong correlations between traits derived from photographic measurements and those obtained from both a global database (AVONET) and our own museum specimens. This preservation of relative size is interesting, given the large variability in bird angles and positions. Although it is not possible to directly estimate the relative bias of field‐based photographic measurements without capturing individuals, independent datasets provide a useful benchmark for comparison. Notably, functional diversity metrics (functional richness and divergence) calculated from the global dataset yielded values comparable to those derived from photographs. In contrast, metrics based on museum specimens differed significantly. These differences are likely driven by changes in the extent of intraspecific trait variability represented in each dataset. The AVONET database incorporates morphological variation across the full geographic range of each species (Tobias et al. 2022), whereas our sampled individuals represent a restricted portion of that range, resulting in lower trait variance. Consequently, functional metrics showed reduced variability in the latter case. Moreover, functional richness exhibited stronger differences than functional divergence, as expected, because it is determined by species at the extremes of the functional space and is therefore more sensitive to limited trait variation and the presence of outliers (Mammola et al. 2021). Therefore, our results based on field photographs suggest that this approach is well suited for comparisons within the same study (e.g., across species or environments), whereas comparisons across studies should be made with caution. In the absence of studies that directly compare photographic and direct measurements on the same individuals, the true magnitude of measurement bias remains uncertain. Nonetheless, our experimental results support the validity of this approach by demonstrating that such bias can be relatively low under specific conditions.

Although our experimental validation included species spanning a broad range of body sizes, the four species evaluated cannot represent the full morphological diversity encountered in bird communities. Therefore, caution is required when extrapolating the observed relationships to taxa with markedly different morphologies, particularly species differing in bill shape, body proportions, plumage structure, or other features that may influence the accuracy of photogrammetric measurements. The field application provided evidence that the approach can generate useful estimates across a substantially larger set of species than those included in the experiment; however, these results should not be interpreted as a validation for all bird taxa. In particular, species with morphologies outside the range represented in the experimental dataset may exhibit different levels of measurement error. Future studies expanding the calibration dataset to include a greater diversity of birds and morphologies will be important to further evaluate the generality of this approach and to identify species or trait combinations for which additional corrections may be required. An additional limitation concerns the estimation of body length. Unlike museum or live‐specimen protocols, which measure total length with the bird fully relaxed (Baldwin et al. 1931), our photogrammetric approach estimates the visible distance from the bill tip to the tail tip in the bird's natural posture. Consequently, this measurement should be regarded as an approximation rather than a direct equivalent of the standard total length. This limitation is particularly relevant because AVONET (Tobias et al. 2022) does not include standard total length measurements, precluding a direct comparison using the conventional definition. Despite this conceptual difference in trait definition, body length estimates showed good agreement with reference values and high repeatability, suggesting that this approximation remains useful for comparative analyses when interpreted appropriately.

An additional consideration when applying photogrammetric approaches in natural settings is the potential for detection and selection biases during field sampling. Photographing free‐ranging birds is inherently more likely to succeed for individuals that remain stationary for longer periods, occur in more exposed positions, or present favorable orientations relative to the camera. Consequently, the subset of individuals captured photographically may not represent a random sample of the local population and could potentially be biased toward particular behavioral, ecological, or morphological characteristics. For instance, pigeons (Picazuro Pigeon *Patagioenas picazuro* , Spot‐winged Pigeon *P. maculosa* , White‐tipped Dove *L. verreauxi* ) and raptors (Roadside Hawk *Rupornis magnirostris* , Snail Kite *Rostrhamus sociabilis* ) typically detect observers at relatively long distances and often flush before a close approach is possible (> 15–20 m). In contrast, some small passerines (e.g., Golde‐crowned Warbler *Basileuterus culicivorus* , Rufous‐collared Sparrow *Zonotrichia capensis* , Tufted Tit‐spinetail *Leptasthenura platensis* ) are comparatively tolerant of human presence and may even approach the observer, allowing photographs to be obtained at much shorter distances (2–4 m). This consideration is particularly relevant when the objective is to quantify intraspecific trait variation or estimate community‐level functional diversity, where non‐random sampling of individuals could influence trait distributions. Although our study focused on evaluating measurement accuracy rather than sampling representativeness, future applications of this approach should consider standardized sampling protocols and, where possible, evaluate whether photographic detectability is associated with individual traits, species characteristics, or ecological attributes. Combining photogrammetric measurements with systematic sampling designs will be important to minimize potential biases when applying this method in community‐level trait studies.

Finally, and although the cosine correction provides a geometrically motivated adjustment for the reduction in projected length caused by specimen orientation, our experimental evaluation indicates that its performance may vary among traits. This likely reflects the fact that the rotation angle of the entire specimen does not necessarily correspond to the effective projection angle of each morphological trait, particularly for structures with complex three‐dimensional geometry. In addition, the correction assumes that the measured structure behaves as a straight segment rotating within a single plane and that its orientation angle is known without error (Leorna et al. 2022). In practice, however, slight inaccuracies in angle estimation, deviations from ideal planar rotation, uncertainty in the location of anatomical landmarks, and small measurement errors can all be amplified by the cosine correction, particularly at larger angles where the correction factor increases rapidly (Huang and Qin 2026). Therefore, the correction should be interpreted as an approximation to reduce orientation‐related bias rather than a complete reconstruction of true three‐dimensional measurements. Nevertheless, applying this correction to field photographs was useful because uncorrected measurements would be expected to systematically underestimate trait sizes when individuals are not oriented parallel to the focal plane, as the projected length decreases in proportion to the cosine of the orientation angle (i.e., cos(*α*) < 1 for all *α* > 0°). Indeed, corrected estimates showed a closer agreement with available benchmark values from AVONET than would be expected from uncorrected measurements, which would have resulted in a stronger systematic underestimation. Thus, while the cosine correction does not completely eliminate orientation‐related errors, it seems to represent a practical adjustment that reduces a major source of bias in community‐level trait measurements obtained from photographs. More generally, photographic estimates should be compared with independence reference datasets to assess their biological plausibility. Importantly, our results suggest that, whenever possible, the structure of interest should be positioned as parallel as possible to the focal plane during image acquisition. Minimizing deviations from this orientation reduces the need for geometric corrections and likely provides more reliable estimates of morphological traits, particularly for structures whose three‐dimensional shape may not follow the rotation of the entire individual.

Overall, the pinhole camera model represents a promising, non‐invasive approach for obtaining morphometric measurements of birds in the field. Despite its conceptual simplicity, this method is highly generalizable and relatively easy to implement, requiring only standard photographic data and basic geometric assumptions. By enabling measurements without capture or handling, it expands the scope of studies to include species that are difficult to trap or sensitive to disturbance. Moreover, its applicability extends beyond morphology, offering new opportunities to quantify traits linked to behavior and ecology, such as prey size, foraging posture, or nesting biology. As such, this approach provides a flexible framework for integrating morphological and ecological data, particularly in contexts where traditional methods are logistically challenging or ethically constrained.

## Author Contributions

**Facundo X. Palacio:** conceptualization (lead), data curation (lead), formal analysis (lead), funding acquisition (lead), methodology (equal), software (lead), writing – original draft (lead), writing – review and editing (lead). **Evaristo Elso:** methodology (equal), writing – review and editing (equal). **Diego Montalti:** conceptualization (supporting), methodology (equal), resources (supporting), writing – review and editing (supporting).

## Funding

Facundo X. Palacio was funded by the American Ornithological Society (Kessel Research Fellowship 2023). Consejo Nacional de Investigaciones Científicas y Técnicas provided partial funding to Facundo X. Palacio and Diego Montalti. Proyecto 11/N980 Facultad de Ciencias Naturales y Museo (UNLP) also provided partial funding to Diego Montalti.

## Conflicts of Interest

The authors declare no conflicts of interest.

## Supporting information


**Appendix S1:** Bird measurements taken from photographs.
**Table S1:** Bias (100 × observed value – “true” value/“true” value) and precision (standard deviation in bias) in bird measurements across species, traits and angles in the experimental setting without applying the trigonometric (cosine) correction for body orientation.
**Table S2:** Bias (100 × observed value – “true” value/“true” value) and precision (standard deviation in bias) in bird measurements across species, traits and angles in the experimental setting after applying the trigonometric (cosine) correction for body orientation.
**Table S3:** Mean absolute percentage error (%) between photographic and reference measurements for each morphological trait. Lower values indicate higher agreement between methods.
**Figure S1:** Bird measurements taken from photographs. A Tufted Tit‐Spinetal ( *Leptasthenura platensis* ), a typical bird from “talares”, is shown. Photo credit: Facundo X. Palacio.
**Figure S2:** Correlations between (angle‐corrected) measurements obtained from photographs and those from AVONET (a–c) and museum specimens (d–g). Points represent species. Dashed lines indicate the 1:1 relationship. Tarsus length (AVONET and museum specimens) and wing length (AVONET and museum specimens) did not differ significantly from the 1:1 line (*p* > 0.05), whereas all other traits showed significant deviations.

## Data Availability

All R codes and data used for the analyses and simulations presented in this article are available at https://doi.org/10.5281/zenodo.21523928.
