# Supplementary material for: Measuring Bird Morphology From Field Photographs
Source: Ecol Evol. 2026 Oct 5;16(10):e74422. doi: 10.1002/ece3.74422 (PMC13638267; doi:10.1002/ece3.74422)
Supplement: Supplementary file 1 [file ECE3-16-e74422-s001.docx]

**Appendix S1:** Bird measurements taken from photographs.

**Table S1.** Bias (100×observed value – ‘true’ value/‘true’ value) and precision (standard deviation in bias) in bird measurements across species, traits and angles in the experimental setting without applying the trigonometric (cosine) correction for body orientation.

| Species | Trait | Angle (°) | Mean bias (%) | Standard deviation |
| --- | --- | --- | --- | --- |
| *Passer domesticus* | Bill length | 0 | 3.61 | 6.92 |
|  |  | 20 | 9.3 | 7.09 |
|  |  | 40 | -4.67 | 8 |
|  |  | 60 | -27.6 | 7.4 |
|  | Wing length | 0 | 1.32 | 5.74 |
|  |  | 20 | -2.88 | 5.66 |
|  |  | 40 | -12.71 | 8.37 |
|  |  | 60 | -23.98 | 11.91 |
|  | Tarsus length | 0 | -5.23 | 11.15 |
|  |  | 20 | 0.46 | 11.43 |
|  |  | 40 | -8.33 | 14.19 |
|  |  | 60 | -26.86 | 20.02 |
|  | Body length | 0 | 5.08 | 5.77 |
|  |  | 20 | 0.9 | 5.86 |
|  |  | 40 | -9.25 | 8.38 |
|  |  | 60 | -24.23 | 10.52 |
| *Turdus rufiventris* | Bill length | 0 | 15.15 | 10.57 |
|  |  | 20 | 12.79 | 12.91 |
|  |  | 40 | 3.49 | 15.04 |
|  |  | 60 | -7.76 | 22.84 |
|  | Wing length | 0 | 8.67 | 7.09 |
|  |  | 20 | 5.42 | 6.87 |
|  |  | 40 | -2.3 | 9.55 |
|  |  | 60 | -13.74 | 11.07 |
|  | Tarsus length | 0 | -11.66 | 9.32 |
|  |  | 20 | -10.04 | 9.2 |
|  |  | 40 | -9.91 | 12.37 |
|  |  | 60 | -11.61 | 9.57 |
|  | Body length | 0 | 4.25 | 8.11 |
|  |  | 20 | 0.55 | 8.49 |
|  |  | 40 | -5.26 | 8.65 |
|  |  | 60 | -11.61 | 10.61 |
| *Colaptes campestris* | Bill length | 0 | 2.45 | 10.39 |
|  |  | 20 | 1.4 | 8.2 |
|  |  | 40 | -10.43 | 9.42 |
|  |  | 60 | -28.49 | 13.61 |
|  | Wing length | 0 | -1.65 | 10.25 |
|  |  | 20 | -0.18 | 10.12 |
|  |  | 40 | 1.01 | 8.97 |
|  |  | 60 | -2 | 6.06 |
|  | Tarsus length | 0 | 7.90 | 10.11 |
|  |  | 20 | -22.49 | 12.03 |
|  |  | 40 | -15.92 | 7.27 |
|  |  | 60 | -15.33 | 6.52 |
|  | Body length | 0 | -5.71 | 10.86 |
|  |  | 20 | -7.88 | 9.89 |
|  |  | 40 | -12.92 | 8.18 |
|  |  | 60 | -19.06 | 14.98 |
| *Milvago chimango* | Bill length | 0 | 9.06 | 11.77 |
|  |  | 20 | 3.21 | 8.24 |
|  |  | 40 | -9.2 | 9.16 |
|  |  | 60 | -26.15 | 10.12 |
|  | Wing length | 0 | -0.98 | 7.01 |
|  |  | 20 | -2.73 | 9.34 |
|  |  | 40 | -5.5 | 13.68 |
|  |  | 60 | -13.99 | 16.85 |
|  | Tarsus length | 0 | 10.83 | 12.85 |
|  |  | 20 | 7.46 | 12.19 |
|  |  | 40 | 4.55 | 10.39 |
|  |  | 60 | 1.22 | 10.23 |
|  | Body length | 0 | 1.34 | 6.9 |
|  |  | 20 | -1.68 | 9.38 |
|  |  | 40 | -6.34 | 11.94 |
|  |  | 60 | -18.36 | 15.69 |

**Table S2.** Bias (100×observed value – ‘true’ value/‘true’ value) and precision (standard deviation in bias) in bird measurements across species, traits and angles in the experimental setting after applying the trigonometric (cosine) correction for body orientation.

| Species | Trait | Angle (°) | Mean bias (%) | Standard deviation |
| --- | --- | --- | --- | --- |
| *Passer domesticus* | Bill length | 0 | 3.61 | 6.92 |
|  |  | 20 | 16.32 | 7.54 |
|  |  | 40 | 24.45 | 10.45 |
|  |  | 60 | 44.8 | 14.79 |
|  | Wing length | 0 | 1.32 | 5.74 |
|  |  | 20 | 3.35 | 6.02 |
|  |  | 40 | 13.95 | 10.93 |
|  |  | 60 | 52.04 | 23.81 |
|  | Tarsus length | 0 | -5.23 | 11.15 |
|  |  | 20 | 6.91 | 12.16 |
|  |  | 40 | 19.67 | 18.53 |
|  |  | 60 | 46.29 | 40.05 |
|  | Body length | 0 | 5.08 | 5.77 |
|  |  | 20 | 7.38 | 6.23 |
|  |  | 40 | 18.46 | 10.94 |
|  |  | 60 | 51.54 | 21.04 |
| *Turdus rufiventris* | Bill length | 0 | 15.15 | 10.57 |
|  |  | 20 | 20.03 | 13.74 |
|  |  | 40 | 35.1 | 19.63 |
|  |  | 60 | 84.48 | 45.68 |
|  | Wing length | 0 | 8.67 | 7.09 |
|  |  | 20 | 12.19 | 7.31 |
|  |  | 40 | 27.54 | 12.47 |
|  |  | 60 | 72.53 | 22.14 |
|  | Tarsus length | 0 | -11.66 | 9.32 |
|  |  | 20 | -4.27 | 9.79 |
|  |  | 40 | 17.61 | 16.15 |
|  |  | 60 | 76.78 | 19.13 |
|  | Body length | 0 | 4.25 | 8.11 |
|  |  | 20 | 7.01 | 9.04 |
|  |  | 40 | 23.68 | 11.29 |
|  |  | 60 | 76.78 | 21.22 |
| *Colaptes campestris* | Bill length | 0 | 2.45 | 10.39 |
|  |  | 20 | 7.91 | 8.72 |
|  |  | 40 | 16.92 | 12.3 |
|  |  | 60 | 43.03 | 27.22 |
|  | Wing length | 0 | -1.65 | 10.25 |
|  |  | 20 | 6.23 | 10.77 |
|  |  | 40 | 31.86 | 11.71 |
|  |  | 60 | 96.01 | 12.12 |
|  | Tarsus length | 0 | 7.90 | 10.11 |
|  |  | 20 | -17.51 | 12.8 |
|  |  | 40 | 9.76 | 9.49 |
|  |  | 60 | 69.34 | 13.04 |
|  | Body length | 0 | -5.71 | 10.86 |
|  |  | 20 | -1.97 | 10.52 |
|  |  | 40 | 13.68 | 10.67 |
|  |  | 60 | 61.87 | 29.96 |
| *Milvago chimango* | Bill length | 0 | 9.06 | 11.77 |
|  |  | 20 | 9.83 | 8.76 |
|  |  | 40 | 18.53 | 11.95 |
|  |  | 60 | 47.7 | 20.23 |
|  | Wing length | 0 | -0.98 | 7.01 |
|  |  | 20 | 3.51 | 9.94 |
|  |  | 40 | 23.36 | 17.85 |
|  |  | 60 | 72.03 | 33.69 |
|  | Tarsus length | 0 | 10.83 | 12.85 |
|  |  | 20 | 14.36 | 12.98 |
|  |  | 40 | 36.48 | 13.57 |
|  |  | 60 | 102.44 | 20.45 |
|  | Body length | 0 | 1.34 | 6.9 |
|  |  | 20 | 4.63 | 9.99 |
|  |  | 40 | 22.26 | 15.59 |
|  |  | 60 | 63.28 | 31.38 |

**Table S3.** Mean absolute percentage error (%) between photographic and reference measurements for each morphological trait. Lower values indicate higher agreement between methods.

| **Source** | **Trait** | **Mean absolute percentage error** |
| --- | --- | --- |
| Museum measurements | Bill length | 20.38 |
|  | Tarsus length | 20.24 |
|  | Wing length | 19.13 |
|  | Body length | 15.38 |
| AVONET | Bill length | 34.96 |
|  | Tarsus length | 17.09 |
|  | Wing length | 19.90 |

**Figure S1.** Bird measurements taken from photographs. A Tufted Tit-Spinetal (*Leptasthenura platensis*), a typical bird from “talares”, is shown. Photo credit: Facundo X. Palacio.

**Figure S2.** Correlations between (angle-corrected) measurements obtained from photographs and those from AVONET (a–c) and museum specimens (d–g). Points represent species. Dashed lines indicate the 1:1 relationship. Tarsus length (AVONET and museum specimens) and wing length (AVONET and museum specimens) did not differ significantly from the 1:1 line (p > 0.05), whereas all other traits showed significant deviations.
